# The Death of Dr. Cushing

**Published:** 1900-06

**Authors:** 


### THE DEATH OF DR. CUSHING.

The death of Dr. George H. Cushing, late of Chicago, but recently resident in California, took place in the latter State, Friday, May 25, 1900. This brief announcement by telegraph of the departure of this prominent man from a life of professional activity is all the information in the writer's possession as this number goes to press. The more extended account of his life work must be deferred until our next number.

